# Influenza Altmetric Attention Score and its association with the influenza season in the USA

**DOI:** 10.12688/f1000research.22127.3

**Published:** 2022-03-08

**Authors:** Saif Aldeen AlRyalat, Khaled Al Oweidat, Mohammad Al-Essa, Khaled Ashouri, Osama El Khatib, Athar Al-Rawashdeh, Abeer Yaseen, Ahmad Toumar, Anas Alrwashdeh

**Affiliations:** 1 University of Jordan, Amman, Jordan; 2 King Hussein Cancer Center, Amman, Jordan

**Keywords:** Influenza, Altmetric, Detection, Vaccine, CDC, Infection

## Abstract

**Background:** Altmetrics measure the impact of journal articles by tracking social media, Wikipedia, public policy documents, blogs, and mainstream news activity, after which an overall Altmetric attention score (AAS) is calculated for every journal article. In this study, we aim to assess the AAS for influenza related articles and its relation to the influenza season in the USA.

**Methods: **This study used the openly available Altmetric data from Altmetric.com. First, we retrieved all influenza-related articles using an advanced PubMed search query, then we inputted the resulted query into Altmetric explorer. We then calculated the average AAS for each month during the years 2012-2018.

**Results** **:** A total of 24,964 PubMed documents were extracted, among them, 12,395 documents had at least one attention. We found a significant difference in mean AAS between February and each of January and March (p< 0.001, mean difference of 117.4 and 460.7, respectively). We found a significant difference between June and each of May and July (p< 0.001, mean difference of 1221.4 and 162.7, respectively). We also found a significant difference between October and each of September and November (p< 0.001, mean difference of 88.8 and 154.8, respectively).

**Conclusion: **We observed a seasonal trend in the attention toward influenza-related research, with three annual peaks that correlated with the beginning, peak, and end of influenza seasons in the USA, according to Centers for Disease Control and Prevention (CDC) data.

## Introduction

In the last few years, a new way to measure the attention brought by journal articles, termed altmetrics (a shortening of “alternative metrics” or “article-level metrics”), was adopted. It was also considered an “alternative” to the conventional citation-based measures. Altmetrics measure the impact and attention of an individual article ^1^. Altmetrics are increasingly recognized tools with an aim to measure the real-time influence of an academic article ^2^. Altmetrics measure the impact of journal articles by tracking social media, Wikipedia, public policy documents, blogs and mainstream news activity, after which an overall Altmetric attention score (AAS) is calculated for every journal article ^3^. Altmetrics have been used to measure the impact of articles on a disease ^4^, or even the impact of article on a whole field ^5^. Altmetric.com is one of the providers of altmetrics and was found to have the best coverage of blog posts, news, and tweets. It pulls data from social media (e.g. Twitter and Facebook), traditional media (e.g. The Guardian and New York Times), blogs for individuals and organizations (e.g. Cancer Research UK), and online reference managers (e.g. Mendeley and CiteULike), policy documents published by official websites (e.g., .gov). The AAS is a quantitative measure of the quality and quantity of attention an output has received, it provides an indicator of the amount of attention a research has received. It weights the amount of attention received by each source based on an algorithm.

Each country has its own influenza detection center; the U.S has the Centers for Disease Control and Prevention (CDC), Europe has the European Influenza Surveillance Scheme (EISS), and Japan has the Infectious Disease Surveillance Center (IDSC) ^6^. The problem of inﬂuenza detection and prediction can be tracked back to Serﬂing’s work in 1963 in epidemiology, which tried to ﬁnd a threshold for inﬂuenza breakout ^7^. Since then, various approaches have been proposed for ﬂu detection and prediction in multiple situations ^7– 9^. A previous project by Google in cooperation with the CDC was able to track in a population based on influenza-related web form queries on the Google search engine ^10^. This approach has paved the way for many new approaches designed using the same concept of using search engines for flu detection in the USA ^11^. In this study, we aim to assess the AAS for influenza related articles. Moreover, we will assess the top articles and journals publishing about influenza in terms of attention they brought.

## Methods

### Search strategy

This study used the openly available Altmetric data by Altmetric.com. Accordingly, this study was exempted from institutional board review IRB approval. We conducted the search on June, 5 ^th^ 2019. To retrieve all articles indexed in PubMed related to influenza, we used MeSH database to extract influenza-related terms, and the following were identified:

- Grippe
- Human Flu
- Human Influenza
- Influenza
- Influenza in Humans

We then searched PubMed database in the following steps:

1- All influenza entry terms mentioned above were used as “MeSH terms”.
2- Language: English.
3- Publication type: Journal articles.
4- Search period: from 1/1/2000 to 31/12/2018.

The following query resulted:

(((((((Grippe[MeSH Terms]) OR Human Flu[MeSH Terms]) OR Human Influenza[MeSH Terms]) OR Influenza[MeSH Terms]) OR Influenza in Humans[MeSH Terms]) AND "english"[Language]) AND ("2000/01/01"[Date - Publication] : "2018/12/31"[Date - Publication])) AND "journal article"[Publication Type]

It is important to note that the filter “Journal article” used in the search query only include original article, and exclude review articles, editorials …etc. We screened the searched results for articles discussing the use of databases to detect influenza in USA.

### Altmetric data

We inputted the resulted search query into Altmetric Explorer, a web-based platform that enables users to browse and report all attention data for every piece of scholarly content. It provides the function of inputting search results already retrieved by the PubMed database ^12^.

Data can be filtered and presented for countries and in specific time periods. We filtered influenza mentions for the USA as a country, to correlate with influenza frequency detected by the CDC, then we measured the AAS for each month in the period from 2012 to 2018, we then calculated the average AAS for each month.

We observed regular monthly mentions of the research output only after January 2012, thus we only included mentions from January 2012 and on. We filtered the search for US mentions only. We collected US mentions of influenza related articles in each month in the years from 2012 to 2018, and we then calculated the average AAS score for each month.

We observe peak AAS scores, which defined as the highest score in a month compared to its previous and next months.

### Statistical analysis

We used SPSS version 21.0 (Chicago, USA) in our analysis. We used mean (± standard deviation) to describe continuous variables (e.g. AAS). We used count (frequency) to describe other nominal variables (e.g. countries). We performed one-way ANOVA followed by Tukey’s post-hoc test to analyze the difference in the mean AAS score between each month, we presented the results in mean difference with 95% confidence interval (CI). All underlying assumptions were met, unless otherwise indicated. We adopted a p-value of 0.05 as the significance threshold.

## Results

A total of 24,964 PubMed documents were extracted. Among them, 12,395 documents had at least one Altmetric point. The total number of mentions for the included documents was 185,744, of which 152,899 were from social media, 20,499 were from news and blogs, 10,608 were from policy and patents, 1,309 were from other sources and 479 were from academic sources. The USA contributed to 28,001 (20.4%) of the total mentions, followed by UK 12,007 (8.8%), and Japan 8,684 (6.3%). The average US mentions for the influenza related articles each month from 2012-2018, and their total mentions are shown in Table 1.

**Table 1.** Average US mentions of influenza-related articles each month in the years 2012 to 2018.

Month	Mean	Total mentions	Std. Deviation
January	959.0805	4274	626.92688
February	1076.5216	4331	614.63388
March	615.8514	3056	276.20525
April	593.2094	3037	288.99383
May	464.7877	2694	170.46013
June	586.1891	2930	271.08380
July	423.5106	2162	196.47219
August	408.3328	2368	141.68444
September	742.6760	3611	346.54126
October	831.4399	4237	441.88501
November	676.6112	3668	253.31778
December	693.9009	3623	369.53838
Total	712.6055	39991	441.73627

**Figure 1.**
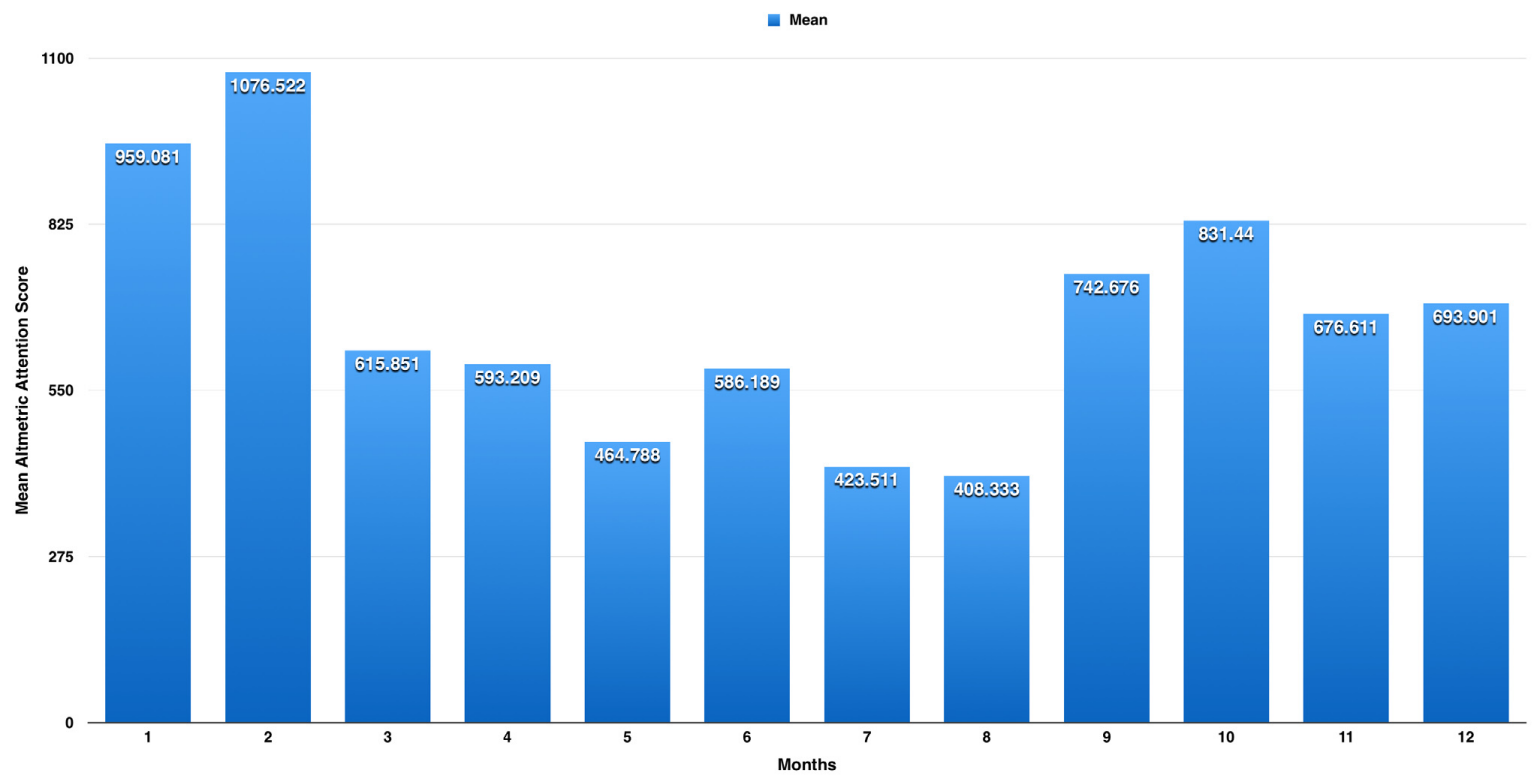
Average Altmetric Attention Score (AAS) for each month from years 2012 to 2018. There are three peaks for the AAS; the highest is observed in February with a mean AAS of 1076.5 (±614.6), the second peak is in October with a mean AAS of 831.4 (±441.9), and the third is in June with a mean AAS of 586.2 (±271.1).

On one-way ANOVA, we found a significant difference between the months (p< 0.001). Following post-hoc analysis, we found a significant difference in mean AAS between February and each of January (p< 0.001, mean difference of 117.4 with 95% CI: 89.7 to 145.2) and March (p< 0.001, mean difference of 460.7 with 95% CI: 430.2 to 491.1). We also found a significant difference between June and each of May (p< 0.001, mean difference of 1221.4 with 95% CI: 87.0 to 155.8) and July (p< 0.001, mean difference of 162.7 with 95% CI: 126.1 to 199.2). We also found a significant difference between October and each of September (p< 0.001, mean difference of 88.8 with 95% CI: 59.6 to 118.0) and November (p< 0.001, mean difference of 154.8 with 95% CI: 125.8 to 183.9). As shown in Figure 1, there are three peaks for the AAS; the highest is observed in February with a mean AAS of 1076.5 (±614.6), the second highest peak is in October with a mean AAS of 831.4 (±441.9), and the third peak is in June with a mean AAS of 586.2 (±271.1).

The journals publishing articles with highest AAS scores were PLOS ONE with a total AAS of 872 for 979 research outputs, followed by Vaccine with 842 for 1015 research outputs, and Influenza & Other Respiratory Viruses with 465 for 465 research outputs. Table 2 shows the journals that published more than 100 influenza-related research during 2019.

**Table 2.** Journals that published more than 100 research output related to influenza during 2019.

Journal	Output
**Vaccine**	1015
**PloS ONE**	979
**Influenza & Other Respiratory Viruses**	507
**Journal of Virology**	350
**Journal of Infectious Diseases**	348
**Emerging Infectious Diseases**	324
**Clinical Infectious Diseases**	291
**BMC Infectious Diseases**	272
**BMC Public Health**	178
**Epidemiology & Infection**	175
**Eurosurveillance**	175
**Human vaccines immunotherapeutics**	171
**Journal of Clinical Virology**	136
**Proceedings of the National Academy of Sciences of the United States of America**	131
**MMWR: Morbidity & Mortality Weekly Report**	128
**The Pediatric Infectious Disease Journal**	123
**Pediatrics**	117
**New England Journal of Medicine**	110
**Infection control and hospital epidemiology (Online)**	109
**PLoS Pathogens**	103
**Journal of Clinical Microbiology**	102
**Scientific Reports**	101

The top research article in terms of AAS is entitled “Infectious virus in exhaled breath of symptomatic seasonal influenza cases from a college community” published in “Proceedings of the National Academy of Sciences of the United States of America” in January 2018, with an AAS of 2927. Table 3 shows research outputs discussing influenza by AAS with an AAS of more than 1000.

**Table 3.** Research outputs with an Altmetric Attention Scores (AAS) of more than 1000 discussing influenza and their respective metrics.

Title	AAS	Journal	Publication date	Citations	Tweets	News mentions	Mendeley readers
Infectious virus in exhaled breath of symptomatic seasonal influenza cases from a college community	2927	Proceedings of the National Academy of Sciences of the United States of America	January 2018	24	2524	250	64
Chasing Seasonal Influenza — The Need for a Universal Influenza Vaccine	2478	New England Journal of Medicine	January 2018	46	1013	309	82
Acute Myocardial Infarction after Laboratory- Confirmed Influenza Infection	2075	New England Journal of Medicine	January 2018	74	1649	148	202
Influenza Vaccine Effectiveness Against Pediatric Deaths: 2010–2014	1889	Pediatrics	April 2017	41	829	249	76
Deposition of respiratory virus pathogens on frequently touched surfaces at airports	1696	BMC Infectious Diseases	August 2018	2	209	246	26
The Japanese Experience with Vaccinating Schoolchildren against Influenza	1686	New England Journal of Medicine	March 2001	611	15299	3	188
Interim Estimates of 2017–18 Seasonal Influenza Vaccine Effectiveness — United States, February 2018	1627	MMWR: Morbidity & Mortality Weekly Report	February 2018	57	870	185	55
1918 Influenza: the Mother of All Pandemics	1391	Emerging Infectious Diseases	January 2006	770	27	231	3
Prevention and Control of Seasonal Influenza with Vaccines	1347	MMWR Recommendations & Reports	August 2016	240	240	164	159
The biggest pandemic risk? Viral misinformation	1346	Nature	October 2018	4	1949	10	33
Potent protection against H5N1 and H7N9 influenza via childhood hemagglutinin imprinting	1278	Science	November 2016	102	148	163	189
An Amphibian Host Defense Peptide Is Virucidal for Human H1 Hemagglutinin- Bearing Influenza Viruses	1167	Immunity	April 2017	16	133	142	86

**Figure 2.**
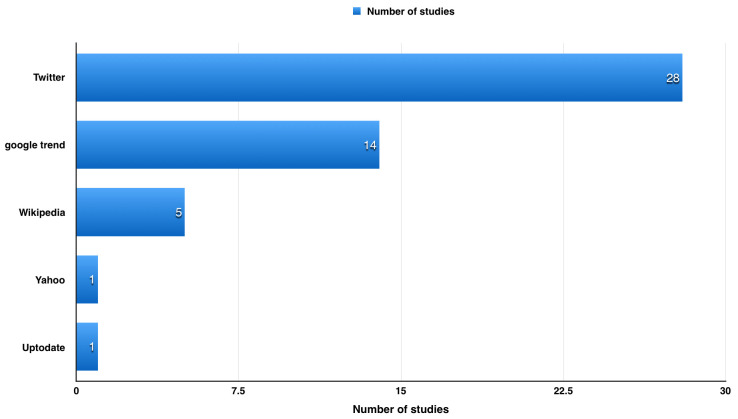
Article discussing the use of websites to detect influenza in USA.

We found around 49 articles discussing the use of websites to detect influenza in USA ( Figure 2).

## Discussion

The research on influenza attracted considerable attention, as measured by the AAS, with the USA the source of the greatest attention. For influenza research from the USA, we observed three peaks for the AAS. The highest peak is observed in February, with a mean AAS of 1076.5 (±614.6), which corresponds to the peak of influenza season as reported by CDC; the second peak is in October with a mean AAS of 831.4 (±441.9), which corresponds to the beginning of the influenza vaccination season; and the third is in June with a mean AAS of 586.2 (±271.1), which corresponds to the end of the influenza season. Almost 10,608 were from policy and patents, representing 5.7% of total attention score, which reflect influenza mentions in official and policy websites (e.g., websites ending with .gov).

Previous studies have used several analytic methods to correlate with influenza season. One of the first studies that brought significant public attention was the one that based its influenza surveillance on Google search engine query data ^9^. in a study co-authored by Google Inc. and CDC researchers. The idea behind this surveillance system was detecting health-seeking behavior in the form of queries to online search engine, where this system managed to estimate weekly influenza activity with only a one-day lag from the CDC actual data. Other studies that used similar estimation techniques followed, where a study by Dugas *et al*. correlated queries to Google search engine with ILI cases reported by emergency departments ^13^. This approach of estimating influenza infection trends based on search engine query was also found to be accurate in other countries, for instance, Europe ^14^, China ^15^, and South Korea ^16^. Other authors also used the Yahoo search engine query to yield similar estimations ^17^. Several studies also used Twitter massages and tweets to detect trends that may correlate with ILI trends as detected by CDC ^11, 18– 21^. Other studies used text mining to extract influenza-related blogs from several web and social media sources ^22^. In another approach, several authors used Wikipedia access logs to achieve accurate, real time estimation of influenza cases ^23, 24^. In a study by Santillana *et al*., the authors combined data from search engines, social media and hospital visits to estimate influenza activity in USA ^25^.

During our literature review, we found around 49 articles discussing the use of websites to detect influenza in USA ( Figure 2). Using search engines as a source of data (e.g. Google and Yahoo) has limited the data provided ^17, 19^, compared to micro-blogging websites (e.g. twitter), which contain more semi-structured metadata enabling a more detailed statistical analysis (e.g. cities, gender, age) ^26^. Several papers proposed different models for detecting flu using Twitter-based methods. Ritterman *et al*. showed that twitter can improve the accuracy of market forecasting by detecting early external events like H1N1 ^27^, followed by another study which used twitter, multiple regression, and document filtering to detect relationship between tweets and national data statistics ^26^. In another study, Broniatowski *et al*. created a new supervised classification model that separates tweets indicating influenza infection from those indicating influenza awareness or concern ^20^.

In general, the interest in publishing about influenza has increased in the recent years ^28^, with USA being the top country in terms of influenza research production ^29, 30^. From the overall influenza research output, influenza vaccine was one of the main topics researched and Journal of Virology and Vaccine journal published the highest number of research articles since 1900 ^29^. We also found that PLOS ONE was the top journal in terms of AAS followed by Vaccine.

With the recent emergence of the coronavirus (COVID-19), severe studies used the altmetric analysis to gain insight about its related publications and public’s response to such new publications ^31^. An altmetric analysis of COVID-19 articles found several factors affecting article’s attention, including the title and how positive were the results ^32^. Another article also showed a higher attention for articles published in high quality journals ^33^. Such higher attention and impact of articles published in higher quality journals might be related to the dedicated social media centers in these journals to publicly promote published articles ^34^. 

Some limitations to the present study need to be taken into account. The search queries in these models are not exclusively submitted by users experiencing influenza-like symptoms, thus the correlations observed might be only meaningful across large populations. In addition, despite strong historical correlations, these systems remain susceptible to false alerts caused by a sudden increase in ILI-related queries. An unusual event, such as a drug recall for a popular cold or flu remedy, announcing a new flu strain, etc., could cause such a false alert ^19^. Disease mentions sometimes depend on social events, which might not be related to disease spread, like holding a conference about flu pandemic. Another limitation to using web-based tools is coverage. Additionally, much of the world is currently excluded from the current systems, which can only process English-language tweets ^20^. Future studies should further assess the validity of our descriptive results by performing sensitivity analysis using the number of articles published each year or proceeding years and correlate AAS score with weekly flu activity data. Moreover, other confirmatory studies may assess the validity of our results by assessing data for influenza in other countries such as Australia and New Zealand, where the influenza seasons are different in timing to those to the USA. 

The use of social media interaction to describe epidemiological studies has been evolving. We observed a seasonal trend in the attention toward influenza-related research, with three annual peaks that correlated with the beginning, peak, and end of influenza seasons in USA, according to CDC data. We believe that analyzing the attention of influenza related research may aid in detecting influenza season’s peaks, which may be a useful tool in areas with limited on-site detection centers. While this study is a descriptive data and its results provide preliminary data, its results should be cautiously interpreted due to the descriptive nature of the study.

## Data availability

### Underlying data

Harvard Dataverse: Altmetric Attention Score for influenza publications in USA. https://doi.org/10.7910/DVN/XCQ8WO ^35^.

This project contains the following underlying data:

- Altmetric - Data.tab (A list of articles found on PubMed that discuss influenza and have at least one Altmetric point)

Data are available under the terms of the Creative Commons Zero "No rights reserved" data waiver (CC0 1.0 Public domain dedication).
