# Supplementary material for: Influenza Altmetric Attention Score and its association with the influenza season in the USA
Source: F1000Res. 2022 Mar 8;9:96. Originally published 2020 Feb 7. [Version 3] doi: 10.12688/f1000research.22127.3 (PMC9021684; doi:10.12688/f1000research.22127.3)
Supplement: Peer review history

# Peer review: Reviewer response for version 3

**DOI:** 10.5256/f1000research.122122.r126660

**Published:** 2022-04-13

**Authors:** Samy A. Azer

**Affiliations:** 1 Department of Medical Education, King Saud University College of Medicine, Riyadh, Saudi Arabia

I thank the authors for submitting an amended version and addressing the points raised. The amended version is acceptable. I have no other comments.

Is the work clearly and accurately presented and does it cite the current literature?

Partly

If applicable, is the statistical analysis and its interpretation appropriate?

Yes

Are all the source data underlying the results available to ensure full reproducibility?

Partly

Is the study design appropriate and is the work technically sound?

Yes

Are the conclusions drawn adequately supported by the results?

Partly

Are sufficient details of methods and analysis provided to allow replication by others?

Partly

Reviewer Expertise:

NA

I confirm that I have read this submission and believe that I have an appropriate level of expertise to confirm that it is of an acceptable scientific standard.

# Peer review: Reviewer response for version 2

**DOI:** 10.5256/f1000research.120137.r120398

**Published:** 2022-02-15

**Authors:** Samy A. Azer

**Affiliations:** 1 Department of Medical Education, King Saud University College of Medicine, Riyadh, Saudi Arabia

Thank you for submitting an amended version of the manuscript. The authors have partly addressed the issues raised by the reviewer. 

 The main problem is limiting the results in the tables to 10 papers or ten journals. What is the scientific basis behind this selection of ten rather than showing a significant number of the results in the tables or appendices? We know that there are errors like these in other publications, but we can not leave such errors repeating. 

 For example, Table 2 does not show the Journals stated in Table 3. The New England Medical Journal is stated three times among the ten mentioned in Table 3, but not in Table 2 even once; the authors can apply this to other journals. The reason is showing small samples of each. 

Is the work clearly and accurately presented and does it cite the current literature?

Partly

If applicable, is the statistical analysis and its interpretation appropriate?

Yes

Are all the source data underlying the results available to ensure full reproducibility?

Partly

Is the study design appropriate and is the work technically sound?

Yes

Are the conclusions drawn adequately supported by the results?

Partly

Are sufficient details of methods and analysis provided to allow replication by others?

Partly

Reviewer Expertise:

Medical informatics. I have published on this area.

I confirm that I have read this submission and believe that I have an appropriate level of expertise to confirm that it is of an acceptable scientific standard, however I have significant reservations, as outlined above.

# Response

**Published:** 2022-03-04

**Authors:** Saif Aldeen AlRyalat

**Affiliations:** University of Jordan, Jordan

Thank you for your productive comments throughout the review process. We agree with the reviewer that choosing “top 10” in general has no scientific basis, rather a haphazard number commonly used by research. Accordingly, we abandoned the “top 10” in this article and reported according to a scientific explanation.

 For table 2, we provided journals that published more than 100 research output related to influenza during 2019.

 For table 3, we provided research outputs with an Altmetric Attention Scores (AAS) of more than 1000 discussing influenza and their respective metrics.

# Peer review: Reviewer response for version 1

**DOI:** 10.5256/f1000research.24402.r91751

**Published:** 2021-10-06

**Authors:** Jafar Kolahi

**Affiliations:** 1 Dental Hypotheses, Isfahan, Iran

1. Keywords should be revised. General terms such as “detection” and “CDC” are not useful.
2. Descriptions about Altmetric resources must be removed from the methods and could be presented in the introduction section.

*“It pulls data from:*

- *Social media (e.g. Twitter and Facebook).*
- *Traditional media (e.g. The Guardian and New York Times).*
- *Blogs for individuals and organizations (e.g. Cancer Research UK).*
- *Online reference managers (e.g. Mendeley and CiteULike).*

*The AAS is a quantitative measure of the quality and quantity of attention an output has received, it provides an indicator of the amount of attention a research has received. It weights the amount of attention received by each source based on an algorithm. "*

1. The main weakness of this manuscript is its statistical methods. The authors aimed to assess the correlation between two longitudinal data “ *We filtered influenza mentions for the USA as a country, to correlate with influenza frequency detected by the CDC*”. Yet do not perform any statistical analysis in this regard. I suggest the usage of https://play95.shinyapps.io/Repeated_Correlation/
2. **Table 2.** To my knowledge, Altmetric institution does not provide Altmetric Attention Score for journals. The only number of mentioned articles and total mentions calculate at the journal level.
3. **Table 3.** Please provide more details for top articles such as the number of Twitter mentions or the number of Mendeley readers, etc.
4. **Figure 2.** This is a finding out of the aims and methods of this study. Please describe methods to reach this data in detail. Why is this important? Presentation of a new findings in the discussion section is not recommended.
5. **152,899 were from social media. **Please describe which social media. Analyze active Twitter accounts in this regard.
6. **10,608 were from policy and patents.** This finding is very important. Please describe policy resources.
7. Please describe the limitations of the study and suggestions for future research in the discussion section.
8. What is the clinical relevance of the findings of this research?
9. Usage of new methods e.g. keyword co-occurrence network analysis or co-citation network analysis is popular among altmetric papers. The authors could consider these methods to increase the values of their research.   

Is the work clearly and accurately presented and does it cite the current literature?

Partly

If applicable, is the statistical analysis and its interpretation appropriate?

No

Are all the source data underlying the results available to ensure full reproducibility?

No

Is the study design appropriate and is the work technically sound?

No

Are the conclusions drawn adequately supported by the results?

Partly

Are sufficient details of methods and analysis provided to allow replication by others?

No

Reviewer Expertise:

Altmetrics

I confirm that I have read this submission and believe that I have an appropriate level of expertise to confirm that it is of an acceptable scientific standard, however I have significant reservations, as outlined above.

# Response

**Published:** 2022-01-08

**Authors:** Saif Aldeen AlRyalat

**Affiliations:** University of Jordan, Jordan

1. Keywords should be revised. General terms such as “detection” and “CDC” are not useful.

Response: Thank you for the suggestion, we edited the keywords.

  

1. Descriptions about Altmetric resources must be removed from the methods and could be presented in the introduction section.

*“It pulls data from:*

- *Social media (e.g. Twitter and Facebook).*
- *Traditional media (e.g. The Guardian and New York Times).*
- *Blogs for individuals and organizations (e.g. Cancer Research UK).*
- *Online reference managers (e.g. Mendeley and CiteULike).*

*The AAS is a quantitative measure of the quality and quantity of attention an output has received, it provides an indicator of the amount of attention a research has received. It weights the amount of attention received by each source based on an algorithm. "*

**Response**: We performed the suggested edits accordingly.

1. The main weakness of this manuscript is its statistical methods. The authors aimed to assess the correlation between two longitudinal data “ *We filtered influenza mentions for the USA as a country, to correlate with* *influenza frequency detected by the CDC* ”. Yet do not perform any statistical analysis in this regard. I suggest the usage of https://play95.shinyapps.io/Repeated_Correlation/

**Response**: Thank you for the suggestion. Our study is mainly a descriptive analysis of the attention scores. We agree with the reviewer that such studies have a strong limitation in its design, which usually does not support inferential statistics. We edited the conclusion and further elaborated on the limitations so that reader keep this limitation in mind upon interpreting the results.

  

1. **Table 2.** To my knowledge, Altmetric institution does not provide Altmetric Attention Score for journals. The only number of mentioned articles and total mentions calculate at the journal level.

**Response**: The Altmetric database analyze the attention score at the article level as the reviewer mentioned, and they accordingly calculate the journal level AAS from their respective articles.

  

1. **Table 3.** Please provide more details for top articles such as the number of Twitter mentions or the number of Mendeley readers, etc.

**Response**: Thank you for the important suggestion. We provided Citations, Tweets, news mentions, Mendeley readers for top articles.

  

1. **Figure 2.** This is a finding out of the aims and methods of this study. Please describe methods to reach this data in detail. Why is this important? Presentation of a new findings in the discussion section is not recommended.

**Response**: We agree with the reviewer, and we added further details in the methods, and we transferred the figure to the results. The articles mentioned in figure 2 were retrieved through the same search described in the methods.

1. **152,899 were from social media. **Please describe which social media. Analyze active Twitter accounts in this regard.

**Response**: We agree with the reviewer on the importance of such details. However, details on twitter accounts can’t be retrieved from the altmetric database. Data regarding other social media mentions and details on calculated attention score was provided in the supplementary data associated with the publication.

1. **10,608 were from policy and patents. **This finding is very important. Please describe policy resources.

**Response**: Thank you. We further elaborated on this result in the introduction and discussion.

  

1. Please describe the limitations of the study and suggestions for future research in the discussion section.

**Response**: We elaborated on the limitations and provided suggestions for future projects:

 “Future studies should further assess the validity of our descriptive results by performing sensitivity analysis using the number of articles published each year or proceeding years and correlate AAS score with weekly flu activity data. Moreover, other confirmatory studies may assess the validity of our results by assessing data for influenza in other countries such as Australia and New Zealand, where the influenza seasons are different in timing to those to the USA.”

  

1. What is the clinical relevance of the findings of this research?

**Response**: We elaborated on the conclusion to further explain the clinical and epidemiological significance of our study, and at the same time, we further explain the inherent limitation of the descriptive nature of our study, and the need for further confirmatory studies.

  

1. Usage of new methods e.g. keyword co-occurrence network analysis or co-citation network analysis is popular among altmetric papers. The authors could consider these methods to increase the values of their research.   

**Response**: We agree with the reviewer on the importance of such methods, and we will work on implementing them in future work.

# Peer review: Reviewer response for version 1

**DOI:** 10.5256/f1000research.24402.r84107

**Published:** 2021-04-29

**Authors:** Samy A. Azer

**Affiliations:** 1 Department of Medical Education, King Saud University College of Medicine, Riyadh, Saudi Arabia

Influenza Altmetric Attention Score and its association with the influenza season in USA 

 Thank you for inviting me to review the above-titled study. The authors have submitted valuable research to the reader of this journal. However, some issues need the authors' attention:

 The title needs to change "USA" to "the USA".

 The abstract, methods need to state, "We filtered influenza mention in the Atmetric data to those related to the USA." 

**Introduction:**

1. The authors must mention after the paper mentioned [Ref 10] that their study is designed along with this principle. They will filter the influenza mention in the Altmetric data obtained to only those related to the USA. This must be mentioned otherwise; a significant criticism could come to the reader's mind. 
2. The writing needs editing for the English.

**Methods:**

1. What do the authors mean by "Journal article"? A journal article can be an article, a perspective, a review, a systemic review, a personal view etc. Which one(s) do they mean?
2. What were the inclusion and exclusion criteria?
3. Altmetric data: "We inputted the resulted search queries into Altmetric Explorer" the statement not clear. Did the authors identify the Altimetric score for each paper for all papers together? How? Did they perform this by themselves or the Altmetric team? 

**Results:** Needs organisation and presenting it to mirror the items in the methods. 

**Discussion:**

1. The study discussion could be strengthened and improved.
2. Language editing is needed.
3. The discussion of findings against other studies in the literature that correlated seasonal findings against the Altmetric scores could be discussed and explaining differences.
4. The limitation of the study - The authors may add they did not study or filter data for influenza in other countries such as Australia and New Zealand, where the influenza seasons are different in timing to those to the USA and tested whether their hypothesis is correct or not. 

**References:** Could be improved and adding other related studies on the Altmetric scores.

Is the work clearly and accurately presented and does it cite the current literature?

Partly

If applicable, is the statistical analysis and its interpretation appropriate?

Yes

Are all the source data underlying the results available to ensure full reproducibility?

Partly

Is the study design appropriate and is the work technically sound?

Yes

Are the conclusions drawn adequately supported by the results?

Partly

Are sufficient details of methods and analysis provided to allow replication by others?

Partly

Reviewer Expertise:

Medical informatics. I have published on this area.

I confirm that I have read this submission and believe that I have an appropriate level of expertise to confirm that it is of an acceptable scientific standard, however I have significant reservations, as outlined above.

# Response

**Published:** 2022-01-08

**Authors:** Saif Aldeen AlRyalat

**Affiliations:** University of Jordan, Jordan

Influenza Altmetric Attention Score and its association with the influenza season in USA 

 Thank you for inviting me to review the above-titled study. The authors have submitted valuable research to the reader of this journal. However, some issues need the authors' attention:

 The title needs to change "USA" to "the USA".

**Response**: Done, thank you.

 The abstract, methods need to state, "We filtered influenza mention in the Atmetric data to those related to the USA." 

**Response**: Done, thank you.

**Introduction:**

1. The authors must mention after the paper mentioned [Ref 10] that their study is designed along with this principle. They will filter the influenza mention in the Altmetric data obtained to only those related to the USA. This must be mentioned otherwise; a significant criticism could come to the reader's mind. 

**Response**: Done, thank you.

  

1. The writing needs editing for the English.

**Response**: We reviewed the article for potential grammatical mistakes, which is also done by the F1000 team.

**Methods:**

1. What do the authors mean by "Journal article"? A journal article can be an article, a perspective, a review, a systemic review, a personal view etc. Which one(s) do they mean?

**Response**: We provided the explanation for this filter. The filter “Journal article” used in the search query only include original article, and exclude review articles, editorials …etc.

  

1. What were the inclusion and exclusion criteria?

**Response**: We provided the details in the methods as follows: “We observed regular monthly mentions of the research output only after January 2012, thus we only included mentions from January 2012 and on. We filtered the search for US mentions only. We collected US mentions of influenza related articles in each month in the years from 2012 to 2018, and we then calculated the average AAS score for each month.”

  

1. Altmetric data: "We inputted the resulted search queries into Altmetric Explorer" the statement not clear. Did the authors identify the Altimetric score for each paper for all papers together? How? Did they perform this by themselves or the Altmetric team? 

**Response**: The Altmetric explorer provide the function of inputting the search results already retrieved by the PubMed database. We provided further details in the methods.

**Results:** Needs organisation and presenting it to mirror the items in the methods. 

**Response**: We’ve relocated part of the results in the methods to provide better organization.

**Discussion:**

1. The study discussion could be strengthened and improved.

**Response**: We further elaborated on the discussion from recently published articles.

  

1. Language editing is needed.

**Response**: We reviewed the article for potential grammatical mistakes, which is also done by the F1000 team.

  

1. The discussion of findings against other studies in the literature that correlated seasonal findings against the Altmetric scores could be discussed and explaining differences.

**Response**:  Thank you for the suggestion, we further elaborated on the discussion. However, the studies already mentioned in the discussion represent the main literature related to altmetric use in epidemiology.

  

1. The limitation of the study - The authors may add they did not study or filter data for influenza in other countries such as Australia and New Zealand, where the influenza seasons are different in timing to those to the USA and tested whether their hypothesis is correct or not. 

**Response**: Thank you for the suggestion, we further elaborated on the study limitations to include this suggestion.

**References:** Could be improved and adding other related studies on the Altmetric scores.

# Peer review: Reviewer response for version 1

**DOI:** 10.5256/f1000research.24402.r65403

**Published:** 2020-06-29

**Authors:** Chintan K Gandhi

**Affiliations:** 1 Department of Pediatrics, Pennsylvania State University College of Medicine, Hershey, PA, USA

I commend the author for carrying out this important study and a very interesting study premise. However, there are a few points that need to be addressed. 

**Major comments:**

 Introduction: The entire second paragraph is irrelevant to the current study. Most of the literature cited in the second paragraph is about flu surveillance activity based on objective hospital data such as hospital deaths, pharmacy use, confirmatory laboratory test. Authors should look for literature about social media use or other similar findings to justify their study. Citation 10 and 11 are appropriate. This paragraph does not support the problem at hand nor justify the study premise.

 Method: The author has written this aim in the introduction, "we aim to assess the AAS for influenza related articles and its relation to the influenza season in USA. Moreover, we will assess the top articles and journals publishing about influenza in terms of attention they brought.". Methods provided can justify the second objective but not the first one. Authors need to carry out sensitivity analysis to correlate AAS score and flu vaccine. For example, CDC releases weekly data about flu activity, author can show the correlation of that particular week with AAS score. In the current method, the author can state that this is a descriptive study of AAS during the flu season in the last couple of years. Author can not fulfill the first objective without sensitivity analysis and correlation with flu activity. 

 Results: This entire paragraph should be in the method section, "We observed regular monthly mentions of the research output only after January 2012, thus we only included mentions from January 2012 and on. We filtered the search for US mentions only. We collected US mentions of influenza related articles in each month in the years from 2012 to 2018, and we then calculated the average AAS score for each month. This is shown in Table 1." Basically, author is describing what they did for the study. I am not sure Table 1 is necessary. You can add standard deviation of table 1 in Figure 1. It is a duplication of information.

 I have trouble understanding results, particularly this section. "As shown in Figure 1, there are three peaks for the AAS; the highest is observed in February with a mean AAS of 1076.5 (±614.6), the second peak is in October with a mean AAS of 831.4 (±441.9), and the third is in June with a mean AAS of 586.2 (±271.1)." What do authors mean by "peak"? As far as I can see from Figure 1, the mean AAS score was highest in February, January, October, September, December in that order. Why the author stated that the second peak was in October and then 3rd peak was in June. It seems figure 1 and the reported result are not correlating. What does "peak" mean? Please clarify. It seems authors trying to fit their data with CDC surveillance data. 

 Author has mentioned this in Method, " We filtered influenza mentions for the USA as a country, to correlate with influenza frequency detected by the CDC, then we measured the AAS for each month in the period from 2012 to 2018, we then calculated the average AAS for each month."

 The cited material here showed that flu activity peaked in February and there is minimal to no activity in April, May, and rest of the summer. If the author is stating that their findings help for flu activity surveillance then accordingly their AAS score should be minimal during those months but instead, the author reported "peak" in June. 

 It is not surprising to see higher AAS during flu season in US. The bottom line is "study findings are overstated." The objective and conclusion of the study are not supported by methods use and reported results, respectively.

 I would advise authors to turn it around and report this study as a descriptive study of AAS during flu seasons.

 Based on the method and results, it is difficult to justify that author's findings will help CDC and/or other surveillance agency to monitor flu activity. For that, authors need 1) to do sensitivity analysis using the number of articles published each year or proceeding years and correlate AAS score (increasing or decreasing) with weekly flu activity data, 2) to choose denominator such as the number of articles published preceding years or something like that rather than just mean AAS score. They need to think out of the box for this, 3) finally, validation of the findings. 

 Discussion: Again, the author mention other surveillance studies but they have mostly reported pharmacy data, hospital, ED visits, and lab data. This should change according to method and results.

 Authors need to make major revisions to the article before it can be accepted for publication.

Is the work clearly and accurately presented and does it cite the current literature?

Partly

If applicable, is the statistical analysis and its interpretation appropriate?

Partly

Are all the source data underlying the results available to ensure full reproducibility?

Yes

Is the study design appropriate and is the work technically sound?

No

Are the conclusions drawn adequately supported by the results?

No

Are sufficient details of methods and analysis provided to allow replication by others?

Partly

Reviewer Expertise:

Trends in outcome data, Surfactant protein A

I confirm that I have read this submission and believe that I have an appropriate level of expertise to confirm that it is of an acceptable scientific standard, however I have significant reservations, as outlined above.

# Response

**Published:** 2022-01-08

**Authors:** Saif Aldeen AlRyalat

**Affiliations:** University of Jordan, Jordan

I commend the author for carrying out this important study and a very interesting study premise. However, there are a few points that need to be addressed. 

**Major comments:**

 Introduction: The entire second paragraph is irrelevant to the current study. Most of the literature cited in the second paragraph is about flu surveillance activity based on objective hospital data such as hospital deaths, pharmacy use, confirmatory laboratory test. Authors should look for literature about social media use or other similar findings to justify their study. Citation 10 and 11 are appropriate. This paragraph does not support the problem at hand nor justify the study premise.

**Response**: Thank you for the suggestion. We omitted the irrelevant part of the introduction, so that only relevant information on influenza detection kept to be more relevant to the current study.

 Method: The author has written this aim in the introduction, "we aim to assess the AAS for influenza related articles and its relation to the influenza season in USA. Moreover, we will assess the top articles and journals publishing about influenza in terms of attention they brought.". Methods provided can justify the second objective but not the first one. Authors need to carry out sensitivity analysis to correlate AAS score and flu vaccine. For example, CDC releases weekly data about flu activity, author can show the correlation of that particular week with AAS score. In the current method, the author can state that this is a descriptive study of AAS during the flu season in the last couple of years. Author can not fulfill the first objective without sensitivity analysis and correlation with flu activity. 

**Response**: We agree with the author on the importance of sensitivity analysis. Due to the absence of such analysis, we modified the aim to be in line with the conducted analysis:

 “In this study, we aim to assess the AAS for influenza related articles. Moreover, we will assess the top articles and journals publishing about influenza in terms of attention they brought”

 Results: This entire paragraph should be in the method section, "We observed regular monthly mentions of the research output only after January 2012, thus we only included mentions from January 2012 and on. We filtered the search for US mentions only. We collected US mentions of influenza related articles in each month in the years from 2012 to 2018, and we then calculated the average AAS score for each month. This is shown in Table 1." Basically, author is describing what they did for the study. I am not sure Table 1 is necessary. You can add standard deviation of table 1 in Figure 1. It is a duplication of information.

**Response**: We agree with the reviewer that this part of results is better fitted within the methods. We made the required changes. Table 1 also mentions the total mentions, which is used in the discussion of our results.

 I have trouble understanding results, particularly this section. "As shown in Figure 1, there are three peaks for the AAS; the highest is observed in February with a mean AAS of 1076.5 (±614.6), the second peak is in October with a mean AAS of 831.4 (±441.9), and the third is in June with a mean AAS of 586.2 (±271.1)." What do authors mean by "peak"? As far as I can see from Figure 1, the mean AAS score was highest in February, January, October, September, December in that order. Why the author stated that the second peak was in October and then 3rd peak was in June. It seems figure 1 and the reported result are not correlating. What does "peak" mean? Please clarify. It seems authors trying to fit their data with CDC surveillance data. 

**Response**: We provided a definition to the “peak” we used in the results section, which is the relatively higher AAS compared to previous and next months. Accordingly, the highest is observed in February with a mean AAS of 1076.5 (±614.6), the second highest peak is in October with a mean AAS of 831.4 (±441.9), and the third peak is in June with a mean AAS of 586.2 (±271.1). The months mentioned by the reviewer has highest absolute values.

 Author has mentioned this in Method, " We filtered influenza mentions for the USA as a country, to correlate with influenza frequency detected by the CDC, then we measured the AAS for each month in the period from 2012 to 2018, we then calculated the average AAS for each month." The cited material here showed that flu activity peaked in February and there is minimal to no activity in April, May, and rest of the summer. If the author is stating that their findings help for flu activity surveillance then accordingly their AAS score should be minimal during those months but instead, the author reported "peak" in June. 

**Response**: We agree that the AAS data is not completely explained by the CDC data related to infection cases. The small peak in June was hypothesized to be related to the end of influenza season. This is further stressed in the limitations.

 It is not surprising to see higher AAS during flu season in US. The bottom line is "study findings are overstated." The objective and conclusion of the study are not supported by methods use and reported results, respectively. I would advise authors to turn it around and report this study as a descriptive study of AAS during flu seasons.

 Response: We agree with the reviewer that the AAS were not completely correlating with the CDC data, and the results should not over-emphasize the findings. We further elaborated on the study limitations and re-worded the conclusion to be in line with these limitations.

**Response**: We further elaborated on the study limitations and conclusion to emphasize on the descriptive nature of the study and its findings.

 Based on the method and results, it is difficult to justify that author's findings will help CDC and/or other surveillance agency to monitor flu activity. For that, authors need 1) to do sensitivity analysis using the number of articles published each year or proceeding years and correlate AAS score (increasing or decreasing) with weekly flu activity data, 2) to choose denominator such as the number of articles published preceding years or something like that rather than just mean AAS score. They need to think out of the box for this, 3) finally, validation of the findings. 

**Response:** Thank you for the great suggestions. We added the suggestions to as what future studies should do to confirm our descriptive results.

 Discussion: Again, the author mention other surveillance studies but they have mostly reported pharmacy data, hospital, ED visits, and lab data. This should change according to method and results.

**Response:** We added further discussion related to our results from recent literature. However, the studies already mentioned in the discussion represent the main literature related to altmetric use in epidemiology.
